# Oral mucosal diseases in children - casuistics from the Department of Dermatology - University of São Paulo - Brazil*

**DOI:** 10.1590/abd1806-4841.20165424

**Published:** 2016

**Authors:** Aline Erthal, Silvia Vanessa Lourenço, Marcello Menta Simonsen Nico

**Affiliations:** 1 Universidade de São Paulo (USP) – São Paulo (SP), Brazil

**Keywords:** Buccal mucosa, Oral mucosa, Xeroderma pigmentosum, Mucocele, Oral ulcer

## Abstract

There are no studies about pediatric oral mucosal diseases performed by dermatologists in Brazil. This study presents the casuistics of oral mucosal diseases in children examined at the Oral Diseases Clinic at the Department of Dermatology - University of São Paulo - Brazil. Cases were retrospectively studied from the hospital records from 2003 to 2015. A hundredsix children have been examined. Commoner lesions examined included mucoceles and aphthae. Rare and difficult cases were also seen and have been published; this clinic is based in a tertiary hospital center that deals mostly with complex cases.

Oral mucosal diseases patients are usually seen by dermatologists, otolaryngologists, gastroenterologists, infectologists and dentists, and other professionals.

The oral diseases clinic of the Dermatology Division of the Hospital das Clínicas of Medical School of University of São Paulo performs per year approximately 900 consultations and assists patients of all ages. It is specialized in clinicopathological diagnosis and treatment of complex cases. It is multidisciplinary, comprising dermatologists, dentists, pathologists and head and neck surgeons. There is a lack of similar services in other Brazilian hospitals.

This clinic is part of the largest clinical and research group for oral diseases linked to a dermatology department in Brazil, and has published 48 scientific articles in indexed journals in the last 10 years; several of these papers refer to children's diseases.^1-6^ So far, we had not conducted a clinical epidemiological study of our pediatric series. There are very few similar studies, none of them carried out by dermatologists.^7^

The casuistics of oral mucosal diseases in children up to 15 years assisted at our clinic was studied. We consulted the visits registry books made between 2003 and 2015 and the medical records were retrieved, noting: age, sex, race, clinical diagnosis, and whether or not biopsies were performed, as well as clinicopathological correlation, comorbidities, laboratory tests conducted, treatments used and evolution.

During the study period, 106 children from zero to 15 years were assisted in the clinic, with the following age distribution: 2 years (two cases), 3 years (5 cases), 4 years (6 cases), 5 years (8 cases), 6 years (5 cases), 7 years (7 cases), 8 years (4 cases), 9 years (9 cases), 10 years (15 cases), 11 years (6 cases), 12 years (12 cases), 13 years (6 cases), 14 years (11 cases) and 15 years (10 cases). There were 57 girls and 49 boys.

The diseases observed are listed in table 1, presenting within the following groups: inflammatory diseases (34 cases), cystic lesions (mucocele) (21 cases), oral manifestations of genodermatoses (15 cases), nonvascular benign tumors (8 cases), vascular lesions (8 cases), infectious diseases (7 cases), cases referred to dentistry (5 cases), trauma (4 cases) and pigmentary lesions (4 cases).

**Table 1:** Diagnosis, number of patients for each diagnosis and treatments and conducts instituted

Diagnosis	N of cases	Established treatments and conducts
**INFLAMMATORY DISEASES**	total: 34	
Mouth ulcer	17	dapsone, colchicine, thalidomide, corticosteroids orally
erythema multiformis	4	corticosteroids orally, acyclovir orally, azathioprine
granulomatous cheilitis	4	dapsone, thalidomide, corticosteroids intralesionally and orally
follicular cheilitis (actinic prurigo)	3	thalidomide
lupus erythematosus	2	referral
pemphigus vulgaris	1	corticosteroids orally
mucous membrane pemphigoid	1	dapsone
lichen planus	1	topical corticosteroids
inflammatory pseudotumor	1	(spontaneous involution)
**CYSTIC LESIONS**	total: 21	
mucocele	21	surgical excision
**GENODERMATOSES**	total: 15	
xeroderma pigmentosum	5	surgical excision
tuberous sclerosis	2	
lipoid proteinosis	2	
dyskeratosis congenita	2	
white sponge nevus	2	
neurofibromatosis	1	
ectodermal dysplasia	1	
**BENIGN TUMORS**	total: 8	
intra oral verrucous nevi	3	
lymphangioma circunscriptum	2	
granular cell tumor	1	surgical excision
rabdomioma	1	
cystic hygroma	1	referral
**VASCULAR LESIONS**	total: 8	
pyogenic granuloma	7	surgical excision
venous lake	1	
**INFECTIOUS DISEASES**	total: 7	
viral warts	5	cryotherapy, electrocoagulation
herpes in the immunocompromised	1	acyclovir intravenously
cutaneous leishmaniasis	1	referral
**PIGMENTARY LESIONS**	total: 4	
melanocytic nevus	2	
constitutional pigmentation	2	
**TRAUMA**	total: 4	
**DENTAL CASES**	total: 5	referral

Blanks: unrealized treatment or monitoring

Surgical procedures were performed in 50 patients: surgical excision in 26, punch biopsy in 20, and cryotherapy with liquid nitrogen in 5.

Drugs systemically administered included: thalidomide (12 patients with aphthae, follicular cheilitis or granulomatous cheilitis), dapsone (4 patients with aphthae, granulomatous cheilitis or mucous membranes pemphigoid), colchicine (6 patients with mouth ulcer), azathioprine (3 patients with granulomatous cheilitis or relapsing erythema multiforme), corticosteroids (4 patients with mouth ulcer, erythema multiforme or pemphigus) and acyclovir (4 patients with herpes simplex or erythema multiforme). Significant adverse events were not observed.

The number of children treated in our clinic during the study period – 106 – was small compared to the large number of consultations (approximately 900 per year). Children up to two years were possibly assisted by pediatricians or pediatric surgeons; otorhinolaryngologists, gastroenterologists and dentists probably examined children of various ages without referring them to dermatology. In addition, the pediatric dermatology group traditionally deals with some diseases such as hemangiomas/vascular malformations and congenital epidermolysis bullosa, without referring patients to the oral diseases clinic. Thus, our study, as well as being influenced by routine flow, probably does not reflect, due to the tertiary characteristic of patients examined, the true incidence and population distribution of pediatric disorders of oral mucosa. Most cases presented difficulties in diagnosis or therapy. Moreover, the essentially dermatological approach possibly also influenced the types of lesions evaluated, no cases of bone or odontogenic lesions were examined. However, given the rarity of several of the lesions examined, several original observations have been recorded.

Aphthous ulcers were the commonest inflammatory lesions observed. Our cases included severe and chronic presentations, both idiopathic as linked to identifiable systemic diseases, always requiring prolonged oral treatment.^2^

More rarely, we observed cases of relapsing mucous erythema multiforme, lichen planus and autoimmune bullous diseases, including a very rare case of mucous membrane pemphigoid and other inflammatory pseudotumor of the tongue.^3,4^

We found 4 cases of cheilitis granulomatosa (Melkersson-Rosenthal syndrome, exceptional in childhood), all were very chronic and resistant to treatment.^5,6^

Mucoceles were the commonest non inflammatory lesions, these occurred most often in the lower lip and less frequently in the ventral tongue (glands of Blandin-Nuhn) and in the floor of the mouth (ranula). Several studies on pediatric mucocele appeared after our publication.^8^ Other interesting benign mucosal tumors observed included verrucous nevus and granular cell tumor.^5^

The observation of oral lesions in several genodermatoses allowed us to formulate some new correlations between their cutaneous and mucosal aspects, establishing a parallelism between them.^9,10^ Of particular interest, we characterized a peculiar lingual lesion in patients with xeroderma pigmentosum that were referred to the oral diseases clinic mainly to have their actinically induced labial changes checked. We noted that several of these children developed a chronic erythematotelangiectatic patch on the anterior border of their tongue. This area is often exposed to sunlight during childhood, hence an alteration very similar to skin lesions may issue, including the propensity for tumor development at this site (Figure 1).^10^

**Figure 1:**
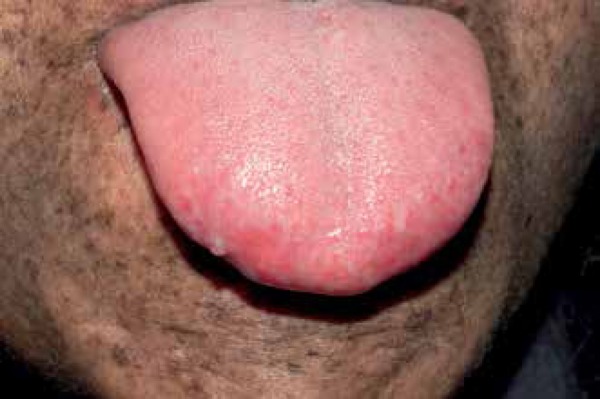
Child with xeroderma pigmentosum. In addition to typical skin changes, an erythematotelangiectatic patch is observed at the anterior portion of the tongue. Squamous cell carcinomas issued at the tip of the tongue in two of four patients with simmilar findings.

Children with dental diseases (dental fistulas, malformations) were addequately reffered to odontology.

The majority of cases with indication of biopsy or surgery were operated under local anesthesia, more often during the first visit.

We believe that the existence of a multidisciplinary clinic in oral diseases in a dermatology service is of great benefit to patients and to professionals involved. The association between dermatologists, dentists and histopatologists allows for a much better comprehension of these diseases.
